# Supplementary material for: Coevolutionary methods enable robust design of modular repressors by reestablishing intra-protein interactions
Source: Nat Commun. 2021 Sep 22;12:5592. doi: 10.1038/s41467-021-25851-6 (PMC8458406; doi:10.1038/s41467-021-25851-6)
Supplement: Supplementary file 1 — Supplementary Information [file 41467_2021_25851_MOESM1_ESM.pdf]

SUPPLEMENTARY INFORMATION

Coevolutionary methods enable robust design of modular repressors by reestablishing intra-protein interactions

Xian-Li Jiang[1,2,8], Rey P. Dimas[3,8], Clement T. Y. Chan[4,5,*], Faruck Morcos[1,6,7,*]

AFFILIATIONS

[1] Department of Biological Sciences, The University of Texas at Dallas, Dallas, TX 75080, USA

[2] Department of Bioinformatics and Computational Biology, The University of Texas M.D. Anderson Cancer Center, Houston, TX 77030, USA

[3] Department of Biology, The University of Texas at Tyler, Tyler, TX 75799, USA

[4] Department of Biomedical Engineering, University of North Texas, Denton, TX 76207, USA

[5] BioDiscovery Institute, University of North Texas, Denton, TX 76207, USA

[6] Department of Bioengineering, The University of Texas at Dallas, Dallas, TX 75080, USA

[7] Center for Systems Biology, The University of Texas at Dallas, Dallas, TX 75080, USA

[8] These authors contributed equally

[*] Correspondence to: tszyanclement.chan@unt.edu (C.T.Y.C.), faruckm@utdallas.edu (F.M.)

SUPPLEMENTARY FIGURES

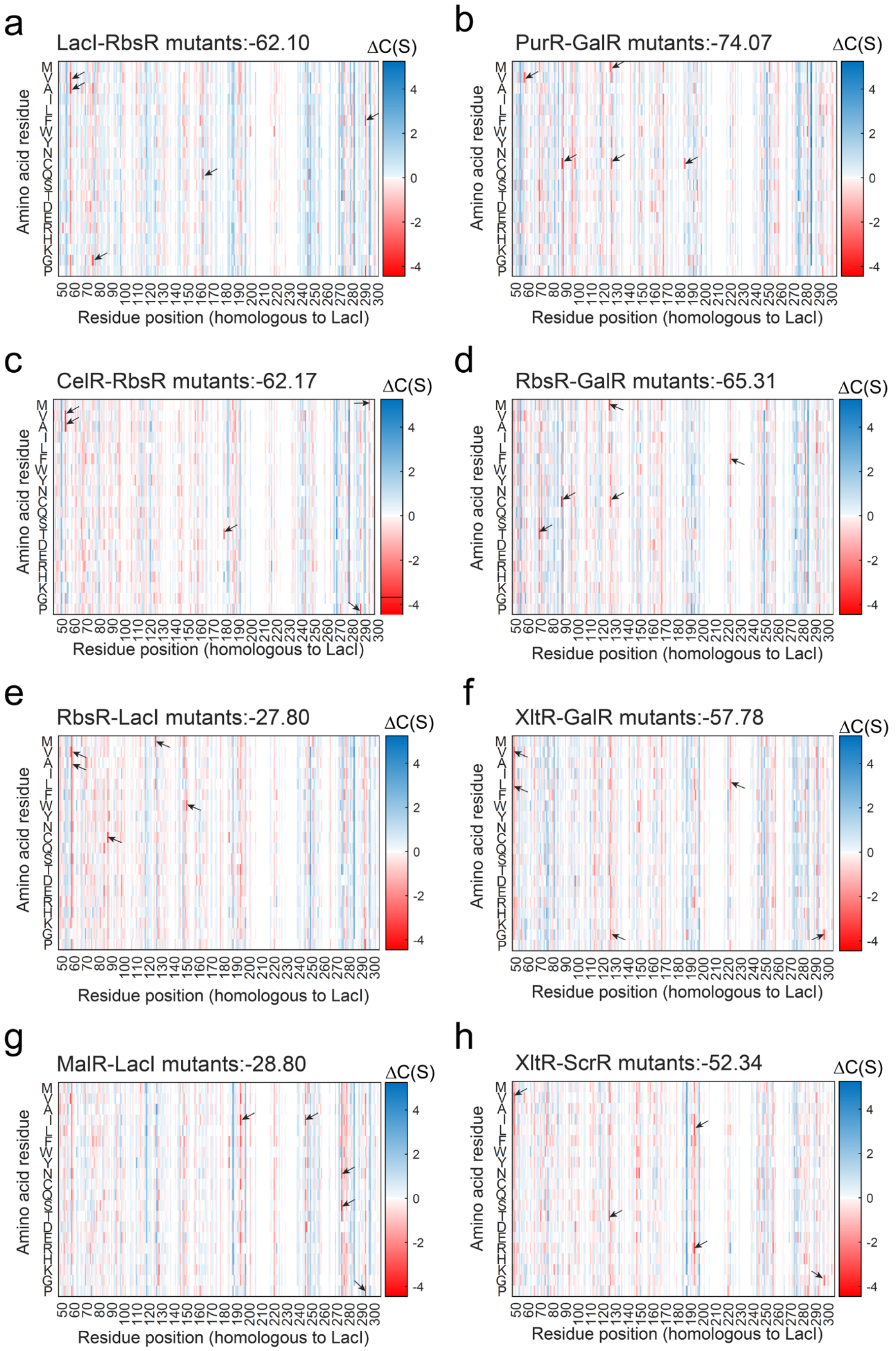

Supplementary Figure 1. Compatibility scores of mutated hybrid repressors. For all poorly functional hybrid involved in this study, including (a) LacI-RbsR (**b**) PurR-GalR, (**c**) CelR-RbsR,(**d**) RbsR-GalR, (**e**) RbsR-LacI, (**f**) XltR-GalR, (**g**) MalR-LacI, and (**h**) XltR-ScrR, we computed the C(S) scores of all possible mutant candidates with a single mutation at the LBM and plotted the ΔC(S) scores relative to the original hybrid repressor. In heatmaps, red represents more favorable scores and blue for less favorable scores. The C(S) score for original hybrid repressor was shown in the title of each panel. Arrows indicates the top 5 mutations.

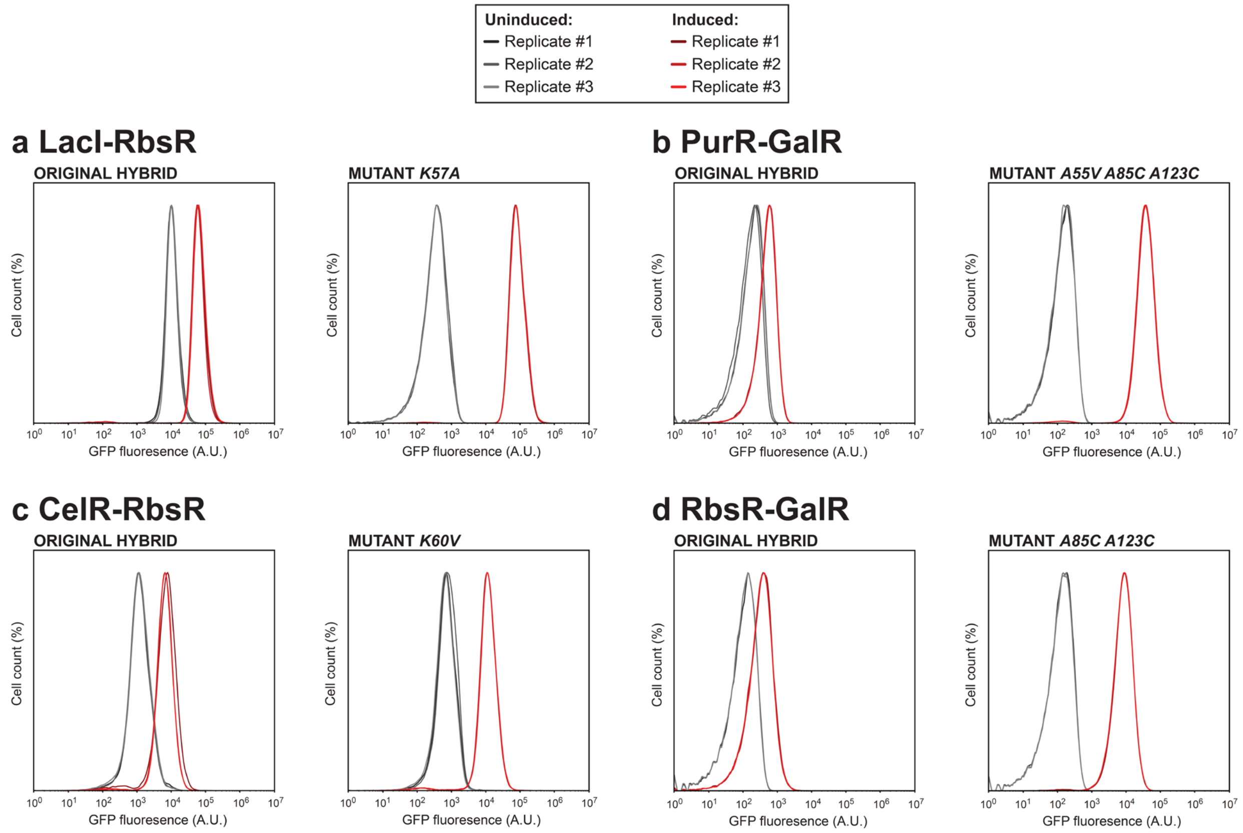

Supplementary Figure 2. Flow cytometric data on characterizing improved hybrid repressors. The distribution of GFP fluorescence (FITC) of each sample are shown for characterization of modified (**a**) LacI-RbsR, (**b**) PurR-GalR, (**c**) CelR-RbsR, and (**d**) RbsR-GalR. In each panel, the two plots illustrate results from the original hybrid repressor (left) and the mutated repressor (right). Each plot shows data from three replicates of uninduced and induced samples. Corresponding quantitative analyses of these data are shown in **Fig. 2**.

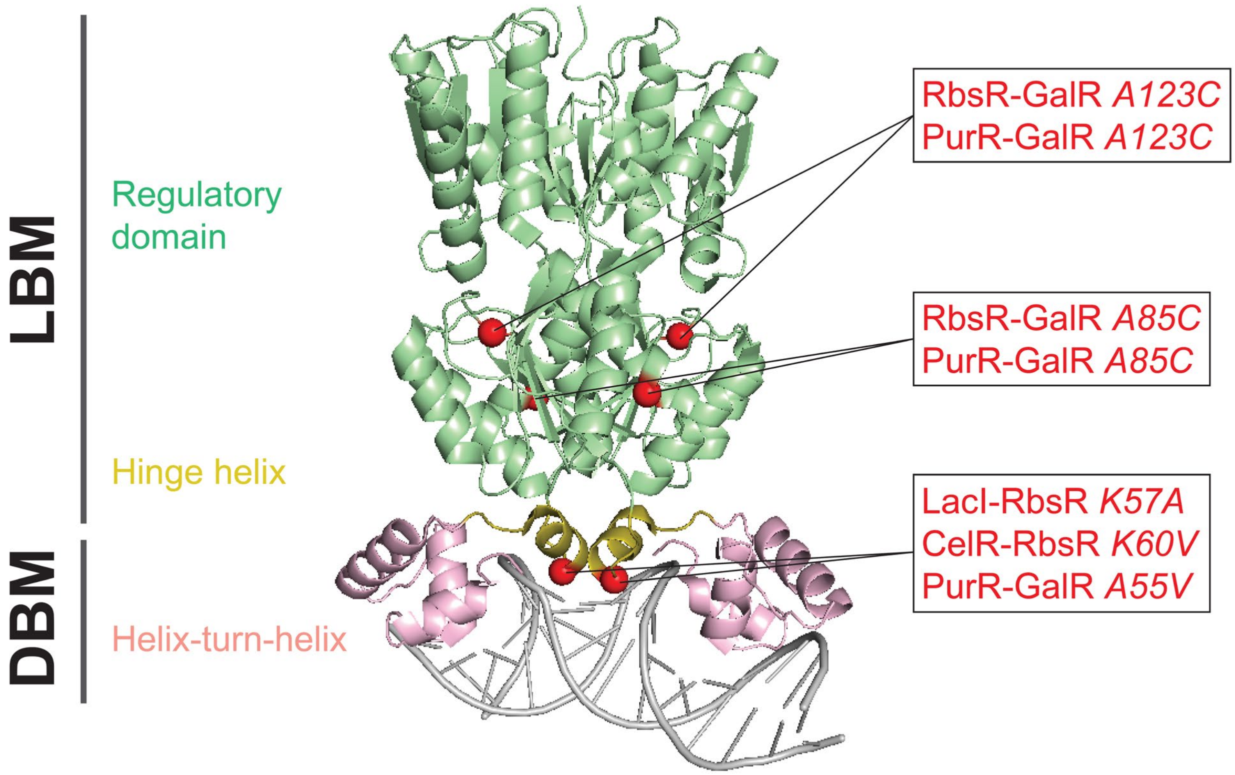

Supplementary Figure 3. Homologous positions on LacI for mutations that rescue hybrid repressors. This is a crystal structure of native LacI in complex with its DNA operator (PDB ID 1EFA). It is a homodimer; the LBM contains a regulator domain (green) and a hinge helix motif (yellow); the DBM only contains the helix-turn-helix motif (pink). Homologous positions of mutations that rescue the four hybrid repressors in **Fig. 2** are highlighted with red spheres.

a

```
XltR-GalR      MPKKSKVTITDIARRVNMTTITVSRALTKPELVKKETLDRILEVARELNYHPNANARALA    60
LacI           ---MKPVTLYDVAEYAGVSYQTVSRVVNQASHVSAKTREKVEAAMAELNYIPNRVAQQLA    57
MalR-LacI      -MNKPQITIKDIARALNVSPSTVSRALKDNPDISKETRDLVHAYAREHNYIPNRVAQQLA    59

XltR-GalR      QQTTETVGLVVGDVSDPFFGAMVKAVEQVAYHTGNFLLIGNGY-HNEQKERQAIEQLIRH    119
LacI           GKQSLLIGVATSSLALHAPSQIVAAIKSRADQLGASVVVSMVERSGVEACKAAVHNLLAQ    117
MalR-LacI      GKQSLLIGVATSSLALHAPSQIVAAIKSRADQLGASVVVSMVERSGVEACKAAVHNLLAQ    119

XltR-GalR      RCAALVVHAKMIPDADL--ASLMKQMPGMVLINRILPGFENRCIALDDRYGAWLATRHLI    177
LacI           RVSGLIINYPLDDQDAIAVEAACTNVPALFL--DVSDQTPINSIIFSHEDGTRLGVEHLV    175
MalR-LacI      RVSGLIINYPLDDQDAIAVEAACTNVPALFL--DVSDQTPINSIIFSHEDGTRLGVEHLV    177
                                   LacI S193
XltR-GalR      QQGHTRIGYLCSNHSISDAEDRLQGYYDALAESGIAANDRLVTFGEPDESGGEQAMTELL    237
LacI           ALGHQQIALLAGPLSSVSARLRLAGWHKYLTRNQIQPIAE--REGDWSAMSGFQQTMQML    233
MalR-LacI      ALGHQQIALLAGPLSSVSARLRLAGWHKYLTRNQIQPIAE--REGDWSAMSGFQQTMQML    235
                                   MalR-LacI S195
XltR-GalR      GRGRNFTAVACYNDSMAAGAMGVLNDNGIDVPGEISLIGFDDVLVSRYVRPRLTTVRYPI    297
LacI           NEGIVPTAMLVANDQMALGAMRAITESGLRVGADISVVGYDDTEDSSCYIPPLTTIKQDF    293
MalR-LacI      NEGIVPTAMLVANDQMALGAMRAITESGLRVGADISVVGYDDTEDSSCYIPPLTTIKQDF    295
                  XltR-GalR A301
XltR-GalR      VTMATQAAELALALADNRPLPEITNVFSPTLVRRHSVSTPSLEA-SHHATSD--------    348
LacI           RLLGQTSVDRLLQLSQGQAVKG-NQLLPVSLVKRKTTLAPNTQTASPRALADSLMQLARQ    352
MalR-LacI      RLLGQTSVDRLLQLSQGQAVKG-NQLLPVSLVKRKTTLAPNTQ-----------------    337
                  LacI G297
XltR-GalR      --------  348
LacI           VSRLESGQ  360
MalR-LacI      --------  337
```

b

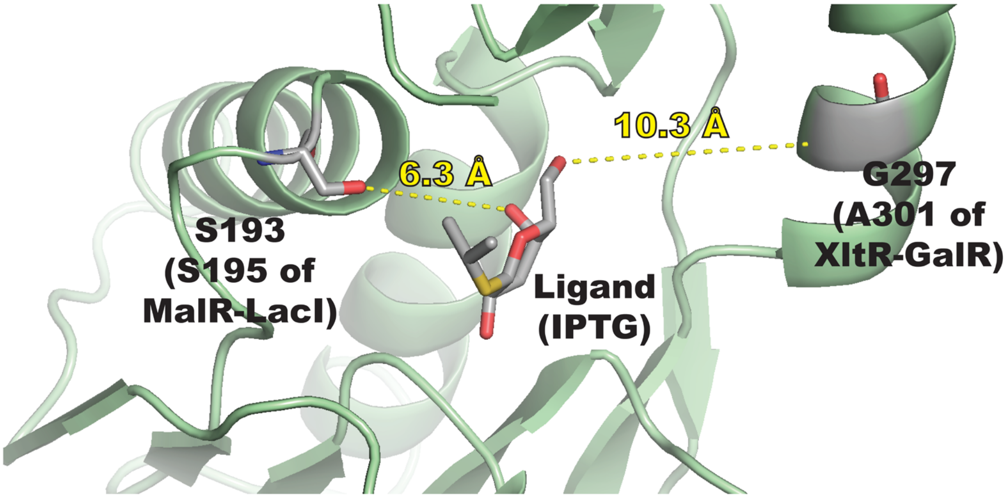

Supplementary Figure 4. Plausible explanation on the low performance of MalR-LacI *S193I*, XltR-GalR *A301G*, and XltR-GalR *E226L/A301G*. As shown in **Figure 3**, these three mutants are improved in both C(S) and SF(S) scores compared to their original hybrid repressors, but their performance remains poor. We then identified the homologous position of MalR-LacI S193 and XltR-GalR A301 in LacI by using sequence alignment (**panel a**). Based upon the crystal structure (**panel b**; PDB ID 1LBH), the ligand, IPTG, is about 6 Å from S191 of LacI, which is the homologous residue of MalR-LacI S193. Since the X-ray crystal structure of GalR has not been solved, knowledge on residues interacting with the ligand in GalR LBM is limited. However, the LacI structure shows that the IPTG molecule is about 10 Å from G297, which is homologous to XltR-GalR A301, suggesting that this residue can be binding to galactose in GalR LBM. These analyses imply that our three mutants are not functioning as expected because their mutations might disrupt ligand binding.

a

RbsR-LacI *V148W*

Residue	Contribution to SF(S)
D127	0.28
A131	0.26
S191	0.25
S189	0.23
L194	0.13

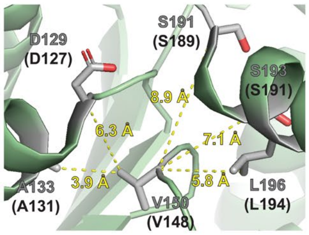

b

RbsR-LacI *N123M*

Residue	Contribution to SF(S)
P74	0.50
M96	0.44
V148	0.38
S67	0.37

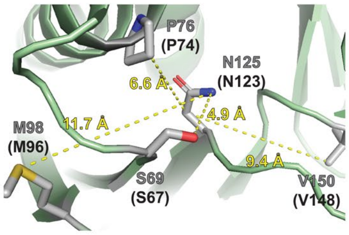

c

RbsR-LacI *A85C*

Residue	Contribution to SF(S)
V92	1.23
G295	0.56
L60	0.36

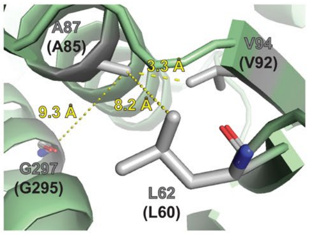

d

RbsR-LacI *G56V*

Residue	Contribution to SF(S)
R49	0.41
A51	0.19

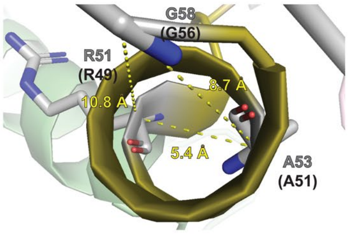

Supplementary Figure 5. Representative illustrations of using SF(S) model and protein structure to investigate mutation interactions. Here we study why our four mutations at RbsR-LacI cannot improve repressor activities, including (**a**) V148W, (**b**) N123M, (**c**) A85C, and (**d**) G56V. In each panel, the table lists the top residues that are the most incompatible with the mutation, based on the SF(S) model; a larger numerical value on the right column means that the residue has a greater contribution to the worsened SF(S) score. The graphical illustrations are from a LacI crystal structure (PDB ID 1EFA), which show these top residues that contribute to the change in SF(S) score. Residue positions in the native LacI are labeled in grey, which are homologous to the residues in RbsR-LacI in the table (labeled in black). The distance between the mutated residue and these residues are shown (yellow).

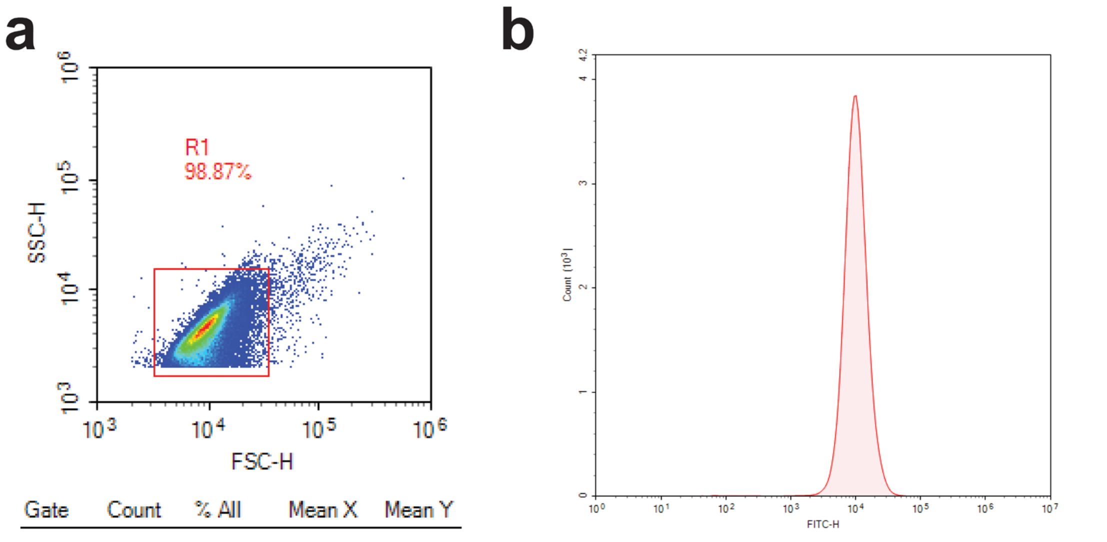

Gate	Count	% All	Mean X	Mean Y
All	57,660	100.00%	10,491	4,467
R1	57,006	98.87%	9,916	4,367

Gate	Color	Count	% Parent	Geom. Mean X
R1	Red	57,006	98.87%	9,378

Supplementary Figure 6. A representative example of the gating strategy for flow cytometric analysis (data from Fig. 2). (**a**) Cells were gated using a forward versus side scatter log area plot, aiming to eliminate multi-cell aggregates. The percentage and total number of cells within the gate is shown. (**b**) The distribution of GFP fluorescence signal from these gated cells are shown in the histogram.
