# Supplementary material for: Coevolutionary methods enable robust design of modular repressors by reestablishing intra-protein interactions
Source: Nat Commun. 2021 Sep 22;12:5592. doi: 10.1038/s41467-021-25851-6 (PMC8458406; doi:10.1038/s41467-021-25851-6)
Supplement: Supplementary file 3 — Description of Additional Supplementary Files [file 41467_2021_25851_MOESM3_ESM.pdf]

Title: Supplementary Data 1.

Description: Sequence of oligonucleotides. This Excel document contains the sequence of all primers used in this study for constructing hybrid repressor genes and Sanger sequencing.
