# Supplementary material for: Coevolutionary methods enable robust design of modular repressors by reestablishing intra-protein interactions
Source: Nat Commun. 2021 Sep 22;12:5592. doi: 10.1038/s41467-021-25851-6 (PMC8458406; doi:10.1038/s41467-021-25851-6)
Supplement: Supplementary file 5 — Reporting Summary [file 41467_2021_25851_MOESM5_ESM.pdf]

nature portfolio

Corresponding author(s): Clement T. Y. Chan and Faruck Morcos

Last updated by author(s): Aug 24, 2021

Reporting Summary

Nature Portfolio wishes to improve the reproducibility of the work that we publish. This form provides structure for consistency and transparency in reporting. For further information on Nature Portfolio policies, see our Editorial Policies and the Editorial Policy Checklist.

Statistics

For all statistical analyses, confirm that the following items are present in the figure legend, table legend, main text, or Methods section.

n/a	Confirmed	
☐	☒	The exact sample size (n) for each experimental group/condition, given as a discrete number and unit of measurement
☐	☒	A statement on whether measurements were taken from distinct samples or whether the same sample was measured repeatedly
☒	☐	The statistical test(s) used AND whether they are one- or two-sided<br>*Only common tests should be described solely by name; describe more complex techniques in the Methods section.*
☒	☐	A description of all covariates tested
☐	☒	A description of any assumptions or corrections, such as tests of normality and adjustment for multiple comparisons
☐	☒	A full description of the statistical parameters including central tendency (e.g. means) or other basic estimates (e.g. regression coefficient) AND variation (e.g. standard deviation) or associated estimates of uncertainty (e.g. confidence intervals)
☒	☐	For null hypothesis testing, the test statistic (e.g. F, t, r) with confidence intervals, effect sizes, degrees of freedom and P value noted<br>*Give P values as exact values whenever suitable.*
☒	☐	For Bayesian analysis, information on the choice of priors and Markov chain Monte Carlo settings
☒	☐	For hierarchical and complex designs, identification of the appropriate level for tests and full reporting of outcomes
☒	☐	Estimates of effect sizes (e.g. Cohen's d, Pearson's r), indicating how they were calculated

Our web collection on statistics for biologists contains articles on many of the points above.

Software and code

Policy information about availability of computer code

Data collection	Data collection of sequences in the LacI family was obtained from Uniprot using custom bash script that contain HMMER 3.2.1 commands.
Data analysis	For analysis of flow cytometric data, geometric means of GFP fluorescence distributions were calculated using the software, NovoExpress 1.4.1. Multiple sequence alignments were built using HMMER 3.2.1 (hmmer.org). Direct Coupling Analysis, the Compatibility Score Model and the Structural Score model were built using custom Matlab R2019b scripts. The code will be available at GitHub: https://github.com/morcoslab/coevolution-compatibility/

For manuscripts utilizing custom algorithms or software that are central to the research but not yet described in published literature, software must be made available to editors and reviewers. We strongly encourage code deposition in a community repository (e.g. GitHub). See the Nature Portfolio guidelines for submitting code & software for further information.

Data

Policy information about availability of data

All manuscripts must include a data availability statement. This statement should provide the following information, where applicable:

- Accession codes, unique identifiers, or web links for publicly available datasets
- A description of any restrictions on data availability
- For clinical datasets or third party data, please ensure that the statement adheres to our policy

All data for the reproduction of the computational result have been deposited in a GitHub repository: https://github.com/morcoslab/coevolution-compatibility, including LacI homologous MSAs collected from Uniprot database, and physical contact residues extracted from a crystal structure of LacI from PDB database under accession code 1LBG. Source data are provided with this paper. All hybrid repressor characterization data generated in this study are provided in the Source Data file.

Field-specific reporting

Please select the one below that is the best fit for your research. If you are not sure, read the appropriate sections before making your selection.

☒ Life sciences ☐ Behavioural & social sciences ☐ Ecological, evolutionary & environmental sciences

For a reference copy of the document with all sections, see nature.com/documents/nr-reporting-summary-flat.pdf

Life sciences study design

All studies must disclose on these points even when the disclosure is negative.

Sample size	The flow cytometer was set to stop collecting data after acquiring 50,000 events. These data were then gated by forward and side scatter to eliminate multi-cell aggregates, which typically excluded less than 10% of the total events (an example to illustrate our gating strategy is provided in Supplementary Fig. 6), and the geometric means of GFP fluorescence distributions were calculated using the software, NovoExpress 1.4.1 (ACEA Biosciences, Inc.). This sample size is sufficient for assessing GFP expression as 10,000 events are enough to generate a smooth normal distribution curve for accurately determining the mean value, as shown in Supplementary Fig. 2.
Data exclusions	No data were excluded from the analysis.
Replication	All biological experiments were performed with three biological replicates. All attempts were consistent.
Randomization	For each experiment, plasmids were transformed into the strain of E. coli cells, which were plated on an agar plate. Three colonies were randomly picked from each plate for culturing and experimental analysis. At OD600 = 0.3, the cell culture from each colony was split into two tubes, one exposed to the inducer molecule and the other one did not. After three hours of incubation, cells were analyzed with flow cytometry; the three samples with inducer exposure were allocated to one group (red circles in each panel of Fig. 2) and the three unexposed samples were in another group (grey circles).
Blinding	Blinding was not relevant to these experiments because cells with particular genetic constituent were required for each experiment.

Reporting for specific materials, systems and methods

We require information from authors about some types of materials, experimental systems and methods used in many studies. Here, indicate whether each material, system or method listed is relevant to your study. If you are not sure if a list item applies to your research, read the appropriate section before selecting a response.

Materials & experimental systems

n/a	Involved in the study	
☒	☐	Antibodies
☒	☐	Eukaryotic cell lines
☒	☐	Palaeontology and archaeology
☒	☐	Animals and other organisms
☒	☐	Human research participants
☒	☐	Clinical data
☒	☐	Dual use research of concern

Methods

n/a	Involved in the study	
☒	☐	ChIP-seq
☐	☒	Flow cytometry
☒	☐	MRI-based neuroimaging

Flow Cytometry

Plots

Confirm that:

☒ The axis labels state the marker and fluorochrome used (e.g. CD4-FITC).

☒ The axis scales are clearly visible. Include numbers along axes only for bottom left plot of group (a 'group' is an analysis of identical markers).

☒ All plots are contour plots with outliers or pseudocolor plots.

☒ A numerical value for number of cells or percentage (with statistics) is provided.

Methodology

Sample preparation	For each sample, a volume of 2 microliters of E. coli culture was added to 200 microliters of phosphate-buffered saline solution. The samples were immediately analyzed with a flow cytometer.
Instrument	ACEA NovoCyte 2030YB flow cytometer (ACEA Biosciences, Inc.)

Software	NovoExpress 1.4.1
Cell population abundance	At least 10,000 events were collected after gating for each measurement.
Gating strategy	Flow cytometry data were gated by forward and side scatter to eliminate multi-cell aggregates. The gating included >80% of data points from living cells acquired by the flow cytometer. All data points within the gating were used to calculate the geometric mean of GFP fluorescence of the cell population.

☒ Tick this box to confirm that a figure exemplifying the gating strategy is provided in the Supplementary Information.
